# Risk stratification in emergency patients by copeptin

**DOI:** 10.1186/1741-7015-12-80

**Published:** 2014-05-16

**Authors:** Kasper Iversen, Jens P Gøtze, Morten Dalsgaard, Henrik Nielsen, Søren Boesgaard, Morten Bay, Vibeke Kirk, Olav W Nielsen, Lars Køber

**Affiliations:** 1 Department of Cardiology, Rigshospitalet, Copenhagen, Denmark; 2 Department of Clinical Biochemistry, Rigshospitalet, Copenhagen, Denmark; 3 Departments of Cardiology and Endocrinology, Hillerød Hospital, Hillerød, Denmark; 4 Department of Cardiology, Bispebjerg Hospital, Copenhagen, Denmark; 5 Department of Cardiology, Frederiksberg Hospital, Copenhagen, Denmark; 6 Department of Oncology, Herlev Hospital, Copenhagen, Denmark; 7 Department of Cardiology, Hillerød Hospital, Dyrehavevej 29, DK-3100 Hillerød, Denmark

**Keywords:** Biomarker, Mortality, Inflammation

## Abstract

**Background:**

Rapid risk stratification is a core task in emergency medicine. Identifying patients at high and low risk shortly after admission could help clinical decision-making regarding treatment, level of observation, allocation of resources and post discharge follow-up. The purpose of the present study was to determine short-, mid- and long-term mortality by plasma measurement of copeptin in unselected admitted patients.

**Method:**

Consecutive patients >40-years-old admitted to an inner-city hospital were included. Within the first 24 hours after admission, a structured medical interview was conducted and self-reported medical history was recorded. All patients underwent a clinical examination, an echocardiographic evaluation and collection of blood for later measurement of risk markers.

**Results:**

Plasma for copeptin measurement was available from 1,320 patients (average age 70.5 years, 59.4% women). Median follow-up time was 11.5 years (range 11.0 to 12.0 years). Copeptin was elevated (that is, above the 97.5 percentile in healthy individuals).

Mortality within the first week was 2.7% (17/627) for patients with elevated copeptin (above the 97.5 percentile, that is, >11.3 pmol/L) compared to 0.1% (1/693) for patients with normal copeptin concentrations (that is, ≤11.3 pmol/L) (*P* <0.01). Three-month mortality was 14.5% (91/627) for patients with elevated copeptin compared to 3.2% (22/693) for patients with normal copeptin. Similar figures for one-year mortality and for the entire observation period were 27.6% (173/627) versus 8.7% (60/693) and 82.9% (520/527) versus 57.5% (398/693) (*P* <0.01 for both), respectively.

Using multivariable Cox regression analyses shows that elevated copeptin was significantly and independently related to short-, mid- and long-term mortality. Adjusted hazard ratios were 2.4 for three-month mortality, 1.9 for one-year mortality and 1.4 for mortality in the entire observation period.

**Conclusions:**

In patients admitted to an inner-city hospital, copeptin was strongly associated with short-, mid- and long-term mortality. The results suggest that rapid copeptin measurement could be a useful tool for both disposition in an emergency department and for mid- and long-term risk assessment.

## Background

An increasing number of patients are referred to emergency departments [1]. Prolonged waiting times and associated crowding have been shown to increase mortality [2,3], and rapid risk stratification is, therefore, a core task in emergency medicine. Identifying patients at high and low risk shortly after admission could help decision-making in the prioritization of patients, treatment, level of observation and post-discharge follow-up. Copeptin is the C-terminal fragment of provassopressin and is presumably co-secreted with arginine vasopressin from the hypothalamus [4]. Copeptin concentrations in plasma increase as a response to physiological stress [5] and have been shown to have prognostic value in several disease entities, such as cardiovascular disease [6-17], head injury [18-20], pulmonary disease [21-24] and shock [25-28], but also in older people with nonspecific complaints [29]. Copeptin could, therefore, be a marker for adverse outcome in unselected patients admitted to a hospital, and its measurement could optimize both short- and long-term risk stratification in an emergency department. The purpose of the present study was to determine short-, mid-, and long-term mortality according to copeptin plasma concentrations in unselected admitted patients. Moreover, the prognostic importance of copeptin measurement in different disease entities was examined.

## Methods

### Patients

Patients were recruited from the Copenhagen Hospital Heart Failure (CHHF) study [30]. The primary aim of CHHF was to investigate if N-terminal pro-brain natriuretic peptide (NT-proBNP) could be used as a screening tool for heart failure in unselected patients admitted to a hospital. Secondary aims were to follow a cohort of adult patients admitted to hospital, irrespective of diagnosis. The CHHF study included all patients >40 years of age admitted to medical or surgical departments at Amager Hospital, Copenhagen, Denmark, between 1 April 1998 and 31 March 1999. In this period 3,644 patients >40-years-old were admitted to the hospital. During the last 10 months of the study blood samples were drawn from patients who gave written informed consent to participate in the study (n = 2,230), The same two dedicated physicians performed the screenings and examinations during the entire study period.

Within the first 24 hours after admission, a structured medical interview was conducted and self-reported medical history was recorded. Additional information from previous hospital admissions was obtained from the patient files. Furthermore, all patients went through a clinical examination and an echocardiographic evaluation of the heart.

After discharge, information regarding primary final diagnosis was collected from patient files. Based on this information, patients were categorized into eight separate disease categories where more rare causes for admission were categorized as ‘other’ (diagnoses of rheumatology (n = 10), nephrology (n = 5), endocrinology (n = 42); and non-specific diagnoses, such as dehydration, social causes, and drug abuse) (see Table 1).

**Table 1:** Patients included and not included in the study

**Baseline variables**	**Included patients**	**Not included patients**	***P*-value**
	**(number = 1,320)**	**(number = 2,309**^**a**^	
**Demographics**			
Age, years	70.5	70.6	0.76
Mean (95% CI)	(69.7 to 71.3)	(70.0 to 71.2)	
Male gender, number (%)	536 (41)	956 (41)	0.61
**Medical history**			
Heart failure, number (%)	160 (12)	244 (13)	0.62
Ischemic heart disease, number (%)	281 (21)	369 (19)	0.15
Previous myocardial infarction, number (%)	132 (10)	224 (12)	0.13
Hypertension, number (%)	349 (27)	455 (24)	0.08
Lung disease, number (%)	255 (19)	365 (19)	0.83
Liver disease, number (%)	39 (3)	58 (3)	0.91
Diabetes, number (%)	141 (11)	220 (11)	0.47
**Discharge diagnoses**			
Heart disease, number (%)	260 (20)	384 (17)	
Orthopedic disease, number (%)	211 (16)	349 (15)	
Gastrointestinal disease, number (%)	168 (13)	287 (12)	
Hematological/oncological, number (%)	85 (6)	154 (7)	0.19
Pulmonary disease, number (%)	85 (6)	150 (7)	
Neurological disease, number (%)	133 (10)	258 (11)	
Infectious disease, number (%)	187 (191)	329 (14)	
Other diseases, number (%)	191 (15)	398 (17)	

^a^Baseline data only available in 2,309 of the 2,324 not included patients (99%).

Information about death was obtained from the Danish Centralized Civil Register using the patients’ unique national Civil Registration Numbers. The Danish Central Office of Civil Registration records the vital status of all residents.

### Blood samples

Blood samples were drawn between 8.00 am and 10.00 am within 24 hours of admission. Samples were centrifuged for 10 minutes at 3,000 rpm. Plasma was stored at –80°C until analysis and only thawed once.

Hemoglobin, C-reactive protein (CRP), creatinine, sodium, and potassium were obtained by standard methods. The glomerular filtration rate was estimated (eGFR) by age, creatinine, and sex using the Modification of Diet in Renal Disease (MDRD) formula [31].

NT-proBNP measurements were taken consecutively during the last 10 months of the study using an ELISA – a two-step sandwich assay with streptavidin-coated microtiter plates (Roche, Basel, Switzerland) [32]. Plasma concentrations of copeptin (ultra-sensitive) were measured in November 2012 on the Kryptor Compact Plus platform (BRAHMS, Hennigsdorf, Germany). The interassay coefficients of variation were 18.3% for 1.4 pmol/L, 6.8% for 9.3 pmol/L and less than 3% for concentrations >18 pmol/L [33]. Reference values of copeptin are based on previously published data, where elevated copeptin was defined as plasma levels above the 97.5 percentile of copeptin concentration in healthy controls (11.3 pmol/L) [4]. In the following, copeptin levels >11.3 pmol/L will be defined as elevated.

### Ethics

The study complied with the Declaration of Helsinki II and was approved by the regional ethics committee of Copenhagen. Following the guidelines for provision of oral and written information, all patients gave written informed consent to participate in the study.

### Statistics

Patients were grouped in three different ways for presentation of data and for statistical analyses: 1, elevated (that is, >11.3 pmol/L = 97.5-percentile in healthy individuals) versus normal copeptin concentrations; 2, quartiles of copeptin; 3, very low (that is, <5-percentile) versus very high (>95-percentile) copeptin concentrations. Associations between categories of variables were measured by the χ^2^ –test or trend test, and Student’s t-test was used for continuous variables. Kaplan-Meier plots were used to illustrate survival curves and Cox proportional hazard model was used for initial univariate comparisons. Multivariable comparisons were performed using a Cox proportional hazard model (fitted by backward elimination using a threshold of *P* <0.1 for elimination) after checking assumptions of proportionality. Cox analyses were made for three-month (short-term) mortality, one-year (mid-term) mortality and mortality during the entire observation period (long-term). Due to the, by default, low mortality during the first week, Cox survival analyses were not made for one-week mortality. Areas under the curve (AUC) were calculated for copeptin in relation to one-week, three-month and one-year mortality, and mortality over the entire study period. C-statistics were calculated for all variables with independent prognostic value. Continuous variables are summarized as means and 95% confidence intervals (CI), while categorical data are summarized as frequencies and percentages. Statistical calculations were done with SPSS version 20.0 (SPSS Inc., Chicago, IL, USA).

## Results

Blood samples were collected from 2,294 patients. Plasma for copeptin measurement was available from 1,320 patients (36% of admitted patients) and these constitute the study population in the present paper. The remaining patients (n = 974) did not have spare plasma for copeptin analysis, but were randomly spread out across the entire study period. Patients included were 70.5 years old on average (95% CI 69.7 to 71.3 years), 59.4% were women and the mean admission time was 6.2 days (95% CI 5.6 to 6.7 days). The patients included in the present analysis were comparable to the ones without plasma samples. There was no difference between included versus excluded regarding baseline variables, comorbidity or discharge diagnoses (Table 1); however, mortality was slightly lower in the included patients (hazard ratio (HR) 0.89, 95% C.I. 0.82 to 0.97).

Median plasma copeptin in the total population was 10.5 pmol/L (range 1 to 725 pmol/L, interquartile range 5.2 to 24.1 pmol/L). The median value was higher in male than in female patients (11.4 pmol/L versus 9.7 pmol/L, *P* = 0.02). Both male and female median values of copeptin were higher than previously reported values in healthy men and women [4] (5.2 pmol/L and 3.7 pmol/L, *P* <0.01 for both). In 47.5% (627/1,320) of the patients, the plasma copeptin level was higher than the previously reported 97.5 percentile in healthy subjects. The 95 percentile, 97.5 percentile and 99 percentile in this population was 89 pmol/L, 125 pmol/L and 211 pmol/L, respectively.

Baseline variables of the patients (demographics, vital parameters, symptoms, medical history and laboratory examinations) in relation to elevated or normal plasma concentration of copeptin are presented in Table 2. Age, systolic and diastolic blood pressure, heart rate, history of heart failure, New York Heart Association (NYHA) class III-IV, ejection fraction, hemoglobin, eGFR, NT-proBNP and left ventricular ejection fraction differed significantly between the patients with elevated plasma copeptin compared to patients with normal plasma copeptin. Copeptin showed a significant but weak correlation with other known markers of mortality (NT-proBNP: r^2^ = 0.14, *P* <0.001; eGFR: r^2^ = 0.10, *P* <0.001; age: r^2^ = 0.04, *P* <0.001; hemoglobin: r^2^ = 0.02, *P* <0.001; potassium: r^2^ = 0.02, *P* <0.001; CRP: r^2^ = 0.01, *P* = 0.03), but not with sodium, *P* = 0.48.

**Table 2:** Baseline data

**Baseline variables**	**Copeptin**	**Copeptin**	***P*-value**
	**≤11.3 pmol/L**	**>11.3 pmol/L**	
	**(number= 693)**	**(number = 627)**	
**Demographics**			
Age, years, mean (95% CI)	66.3 (65.1-67.2)	75.3 (74.3-76.3)	<0.01
Male gender, number (%)	264 (38)	272 (43)	0.05
**Vital signs**			
Systolic blood pressure, mmHg, mean (95% CI)	150 (148-153)	147 (144-149)	0.01
Diastolic blood pressure, mmHg, mean (95% CI)	86 (84-87)	81 (80-83)	<0.01
Heart rate, b/min, mean (95% CI)	86 (84-97)	87 (86-89)	<0.01
**Medical history**			
Heart failure, number (%)	58 (8)	102 (16)	<0.01
Ischemic heart disease, umber (%)	136 (20)	145 (23)	0.11
Previous myocardial infarction, number (%)	63 (9)	69 (11)	0.20
Hypertension, number (%)	183 (27)	166 (27)	0.92
Lung disease, number (%)	144 (21)	111 (18)	0.18
Liver disease, number (%)	20 (3)	19 (3)	0.86
Diabetes, number (%)	65 (9)	76 (12)	0.12
NYHA III-IV, n (%)	32 (5)	72 (11)	<0.01
**Laboratory examinations**			
Sodium mmol/L, mean (95% CI)	137 (137-137)	137 (136-137)	0.06
Potassium, mmol/L, mean (95% CI)	4.0 (3.9-4.0)	4.0 (3.9-4.0)	0.61
Hemoglobin, mmol/L, mean (95% CI)	8.4 (8.3-8.4)	7.9 (7.8-8.0)	0.01
C-reactive protein, mg/L, mean (95% CI)	656 (532-780)	839 (721-956)	0.05
e-GFR, ml/min, mean (95% CI)	92 (90-95)	74 (72-77)	0.01
NT proBNP, pmol/L, mean (95% CI)	147 (125-164)	499 (426-572)	0.01
Ejection fraction, %, mean (95% CI)	60.3 (59.5-61.0)	57.0 (56.0-57.9)	0.01

b/min, beats per minute; CI, confidence interval; eGFR, estimated glomerular filtration rate; NT proBNP, N-terminal pro-brain natriuretic peptide; NYHA, New York Heart Association.

Median follow-up time was 11.5 years, (range 11.0 to 12.0 years). Complete follow-up was possible for all except one patient after one year and for all except eight patients during the entire observation period. The reason for missing information was emigration. All nine patients were censored at time of immigration. One-week mortality was 1.4% (18/1,320), three-month mortality was 8.6% (113/1,320), one-year mortality was 17.7% (233/1,320 patients), and 69.3% (915/1,320 patients) died during the entire study period.

**Figure 1:**
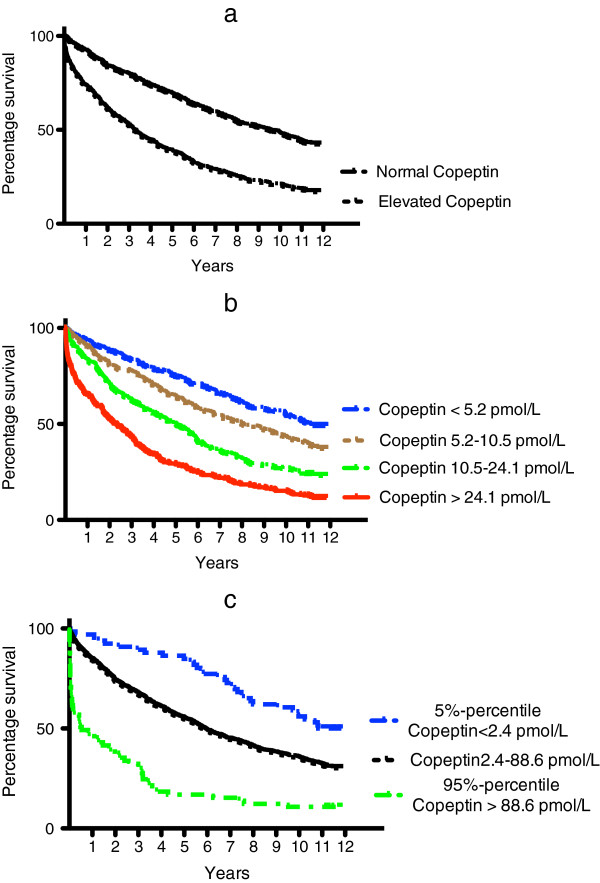
Kaplan-Meier plots for patients with elevated versus normal copeptin (a), quartiles of copeptin (b), very high versus very low copeptin (c).

Figure 1 shows Kaplan-Meier curves for survival of patients with normal and elevated levels of copeptin (**a**), for quartiles of copeptin (**b**), and for patients with very low (<5-percentile) and very high copeptin (>95-percentile) (**c**). Mortality within the first week, three-month mortality, one-year mortality, and mortality for the entire observation period for different groupings of copeptin appear in Table 3. Copeptin was strongly related to mortality independent of grouping of copeptin and observation time. However, the highest differences were seen for very high versus very low copeptin and short observation time.

**Table 3:** Unadjusted mortality rates according to copeptin levels on short-, mid- and long-term mortality

**Time period**	**Reference level**		**Quartiles**		**5th-percentile versus**		***P*-value**
					**95th-percentile**		
	**Normal**	**Elevated**	**Lowest**	**Highest**	**5th-percentile**	**95th-percentile**	
One week	0.1%	2.7%	0 %	4.2%	0%	10.6%	*P* <0.01 for all
0-3 months	3.2%	14.5%	3.0%	20.0%	1.5%	43.9%	*P* <0.01 for all
0-12 months	8.7%	27.6%	7.6%	34.8%	3.0%	54.5%	*P* <0.01 for all
Entire study	57.5%	82.9%	50.9%	88.2%	50.0%	89.4%	*P* <0.01 for all

Using multivariable Cox regression analyses, we examined the prognostic value of copeptin in relation to elevated values of copeptin. All 20 variables from Table 3 were tested as covariates. After backward elimination, NT-proBNP (log-transformed), gender, age, liver disease, potassium and hemoglobin remained in the fully adjusted model as being significantly associated with mortality.

**Figure 2:**
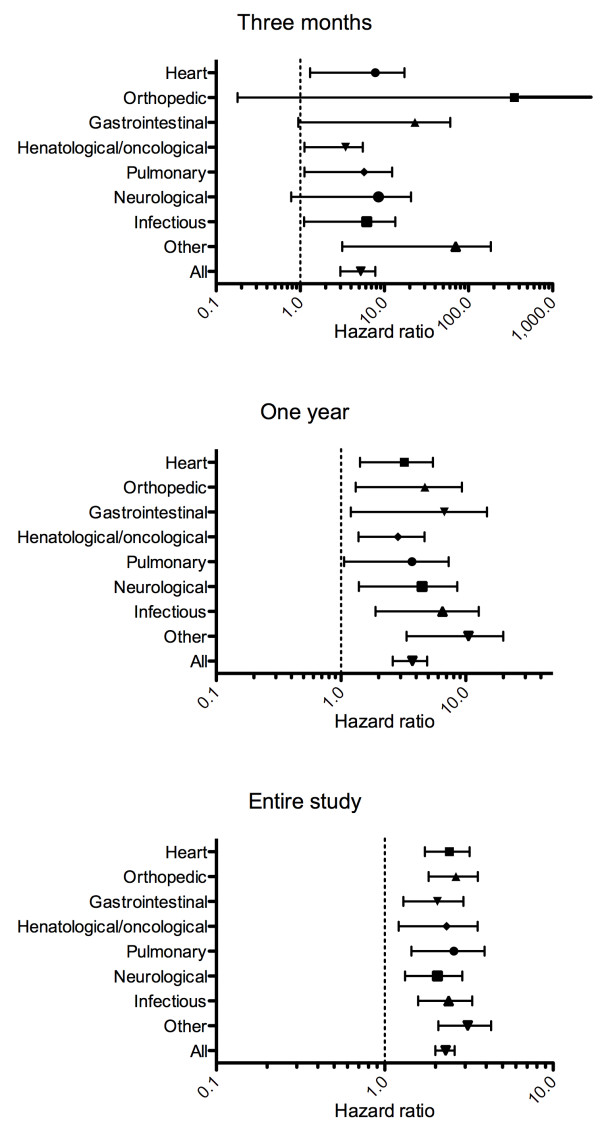
Unadjusted hazard ratios associated with elevated copeptin on three month-, one year-, and entire study mortality in different disease entities.

**Figure 3:**
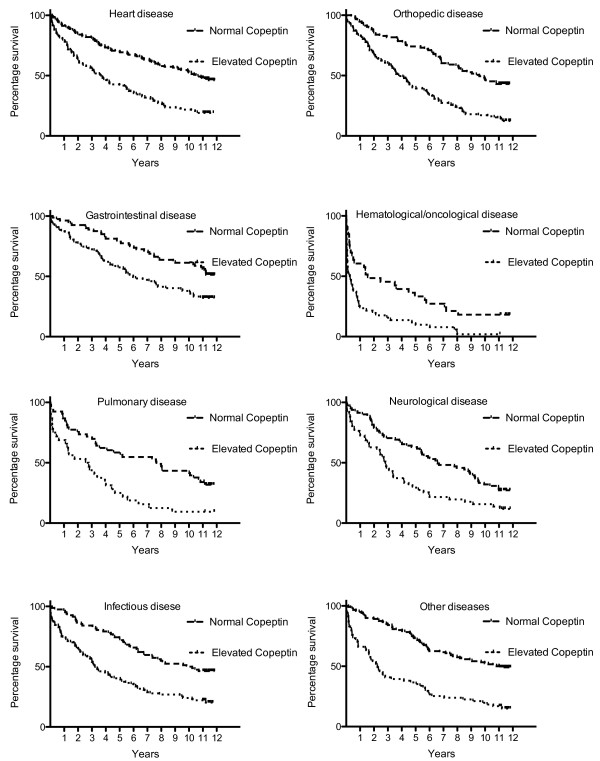
Kaplan-Meier curves illustrating the prognostic value of having elevated copeptin for different disease entities.

Table 4 shows that elevated copeptin, quartiles of copeptin and copeptin levels above the 95^th^-percentile were significantly and independently related to short-, mid- and long-term mortality in the entire population when the above-mentioned variables were included in the model.HRs for patients with elevated copeptin in the different disease categories are shown in Figure 2. The HR for death was highest short-term, but almost all HRs were significantly different from 1. Figure 3 illustrates Kaplan-Meier curves for overall survival according to discharge diagnosis and elevated copeptin (>11.3 pmol/L).

**Table 4:** Unadjusted and adjusted hazard ratios associated with copeptin levels on short-, mid-, and long-term mortality

	**Unadjusted**			**Adjusted**^**a**^		
	**hazard ratio (95% CI)**			**hazard ratio (95% CI)**		
	**3 months**	**First year**	**Total study period**	**3 months**	**First year**	**Total study period**
**Quartiles**						
Quartile 1 (ref.) <5.2 pmol/L	1	1	1	1	1	1
Quartile 2 5.2-10.5 pmol/L	1.10 (0.47-2.59)	1.33 (0.79-2.24)	1.39 (1.13-1.70)	0.70 (0.29-1.69)	0.87 (0.50-1.50)	1.01 (0.81-1.26)
Quartile 3 10.5-24.1 pmol/L	2.66 (1.28-5.52)	2.53 (1.58-4.04)	2.14 (1.76-2.61)	1.40 (0.66-2.96)	1.27 (0.74-2.08)	1.21 (0.98-1.51)
Quartile 4 >24.1 pmol/L	7.40 (3.80-14.39)	5.62 (3.65-8.66)	3.52 (2.91-4.27)	2.48 (1.19-5.15)	1.95 (1.20-3.17)	1.65 (1.33-2.06)
**Elevated copeptin (>11.3 pmol/L)**						
Normal copeptin (ref.)	1	1	1	1	1	1
Elevated copeptin	4.90 (3.08-7.81)	3.61 (2.70-4.85)	2.28 (2.00-2.60)	2.44 (1.47-4.08)	1.85 (1.33-2.58)	1.36 (1.17-1.57)
**95%-percentile vs. 5%-percentile**						
5%-percentile (ref.) <2.4 pmol/L	1	1	1	1	1	1
95%-percentile	40.43 (5.51-296.90)	29.36 (7.07-121.99)	5.41 (5.53-8.29)	7.09 (0.92-54.86)	4.99 (1.16-21.50)	1.60 (1.00-2.58)
**Per 1 increase in log copeptin**	6.66 (4.63-9.57)	4.24 (3.29-5.47)	2.76 (2.41-3.15)	3.24 (2.07-5.08)	1.96 (1.43-2.68)	1.52 (1.29-1.79)

^a^N-terminal pro-brain natriuretic peptide (log-transformed), gender, age, liver disease, potassium, and hemoglobin remained in the model. CI, confidence interval.

Receiver operating characteristic (ROC) analyses show that the AUC for copeptin to predict one-week, short-, mid- and long-term mortality was 0.84 (95% CI 0.77 to 0.92), 0.75 (95% CI 0.70 to 0.80), 0.71 (95% CI 0.67 to 0.74) and 0.70 (0.67 to 0.73), respectively. Comparing a model with age, gender and comorbidity (model 1) to a model containing all variables included in the final Cox model (that is, NT-proBNP (log-transformed), gender, age, liver disease, potassium and hemoglobin) (model 2) and to the fully adjusted model with copeptin added (model 3), we found a significant increase in AUCs for one-week mortality (0.70 (95% C.I. 0.65 to 0.76) versus 0.80 (95% CI 0.73 to 0.88) versus 0.85 (95% CI 0.77 to 0.93), *P* = 0.04) and for three-month mortality (0.72 (95% C.I. 0.70 to 0.75) versus 0.77 (95% CI 0.73 to 0.81) versus 0.79 (95% CI 0.75 to 0.83), *P* = 0.01), respectively. There was no significant effect on AUCs of adding copeptin to the fully adjusted model for one-year mortality or for the entire observation period.

## Discussion

To the best of our knowledge, this study is the first to determine the prognostic value of copeptin measurement in unselected patients ≥40- years old admitted to both medical and surgical departments. Copeptin measurement had a strong and independent prognostic importance for all patients and seems not to be related to any specific diagnosis. Patients with low copeptin concentrations had an excellent short-term prognosis with no fatal outcomes within the first week and only very few deaths within three months. On the other hand, high copeptin concentrations identified a subgroup of patients with a very high risk of short-term mortality.

### Previous studies

The cut-off level for elevated copeptin in the present analysis is based on the 97.5 percentile from the original assay article(4). Recently Keller *et al*. published a slightly higher 97.5 percentile (that is, 13 pmol/L) based on data from the Gutenberg health study [34]. This population is however not a healthy population but a random population sample also including patients with a broad spectrum of different conditions and diseases. This might explain the slightly higher cut-off value in this population and is the reason why we chose to use the original published cut-off values for elevated copeptin.

Our findings corroborate and extend previous studies investigating the prognostic role of copeptin in different settings. In patients with ischemic heart disease, copeptin has been shown to have a high prognostic value with adjusted HRs comparable to the results from the present study [6,12]. Similar results have been reported from studies of patients with heart failure where copeptin seems to be an even stronger predictor for outcome than natriuretic peptides [7,17]. In patients with neurological disease, such as ischemic stroke [10,14,15], intracereberal hemorrhage [18,20] and traumatic head injury [19], copeptin seems to be an excellent marker for both neurological outcome and short- and mid-term mortality. The same prognostic value of copeptin is seen in patients with lung infections [21,23] and chronic obstructive lung disease [25,24,35]. Also, in non-disease specific conditions, such as shock [25-28,36], and in surgical patients [37] copeptin seems to be a valuable marker for outcome. Recently, Nickel and coworkers have published a study describing the short-time prognostic role of copeptin in patients presenting in the emergency department with non-specific complaints. In accordance with the present study they found a strong and independent prognostic value of copeptin [29].

### Strengths and limitations

The strengths of this study are the size of the cohort, its prospective and relative unselected nature, and the long follow-up period. A cohort of unselected patients admitted to a hospital is unique and makes it possible to test the role of copeptin under various medical and surgical conditions. The heterogeneity of the different disease subgroups and limited number of patients within each group does not allow firm conclusions but serves to generate further hypotheses. Only all-cause mortality was registered. Causes of death and admissions would have been other interesting endpoints, but their validity is suboptimal in the registries. The study was a single center study and this could potentially cause problems with generalizability. This concern is, however, partly countered by the fact that the hospital is placed in a part of Copenhagen that includes both high income and low income areas as well as inner city and surburban /country areas. Blood samples were stored for 12 years at –80°C, which could introduce a problem with degradation. However, stability analyses of copeptin have shown that copeptin is a very stable peptide, and no degradation is seen after several cycles of freeze/thaw or after as much as seven days at room temperature [4,33]. Even if there was some degree of degradation of copeptin over the 12 years, this does not pose a problem in the overall interpretation concerning risk. The measured concentrations are most likely underestimated and would have been slightly higher if measured in fresh samples.

Blood was available from only 53% of the originally included patients, the main reason being a lack of spare plasma after blood had been used for other purposes. However, we did not identify any systematic reason for this selection, and there were no differences in baseline variables or mortality between included and not included patients. Some selection bias in the original cohort was inevitable due to missing informed consent from confused, mentally disabled and frail patients. The mean age of the population in the study is high (74 years) and there was a high prevalence of comorbidity. Therefore, the results of this study cannot without caution be extrapolated to younger or non-hospitalized populations.

### Clinical implications

The results of this study suggest that copeptin could be a very useful tool for disposition in an emergency department. Knowing that patients had a seven-day mortality of more than 10% or a one-year mortality of less than 3% might influence the clinician’s decisions regarding observation and treatment in the emergency department. Studies evaluating an intervention (that is, observation, admission or discharge) following measurement of copeptin could show if the findings in the present study could be transformed to a changed and improved clinical practice.

## Conclusions

In patients ≥40 years of age admitted to an inner-city hospital, copeptin in plasma was strongly associated with short-, mid- and long-term mortality. Having very high or very low copeptin identifies a subgroup of patients, respectively, with very poor and very good short-term prognosis. Copeptin seems to be valuable in the most frequent disease entities.

## Abbreviations

AUC: area under the curve; AVP: arginine vasopressin; CHHF: Copenhagen Hospital Heart Failure; CI: confidence interval; CRP: C-reactive protein; eGFR: estimated glomerular filtration rate; ELISA: enzyme-linked immunosorbent assay; HR: hazard ratio; NT-proBNP: N-terminal pro-brain natriuretic peptide.

## Competing interests

The authors declare that they have no competing interests.

## Authors’ contributions

MB and VK collected data and critically revised the manuscript. JPG, SB, MD, OWN and HN participated in drafting of the manuscript and interpretation of the data and critically revised the manuscript. LK participated in drafting of the manuscript, data analyses and interpretation of the data and critically revised the manuscript. KI wrote the first draft of the manuscript, performed the analyses and interpretation of the data. All authors read and approved the final manuscript.

## Pre-publication history

The pre-publication history for this paper can be accessed here:

http://www.biomedcentral.com/1741-7015/12/80/prepub
